# A bioenergetics evaluation of temperature‐dependent selection for the spawning phenology by Snake River fall Chinook salmon

**DOI:** 10.1002/ece3.4353

**Published:** 2018-09-12

**Authors:** John M. Plumb

**Affiliations:** ^1^ Columbia River Research Laboratory Western Fisheries Research Center U.S. Geological Survey Cook Washington

**Keywords:** bioenergetics, spawning salmon, temperature‐dependence

## Abstract

High water temperatures can increase the energetic cost for salmon to migrate and spawn, which can be important for Snake River fall‐run Chinook salmon because they migrate great distances (>500 km) at a time when river temperatures (18–24°C) can be above their optimum temperatures (16.5°C). Average river temperatures and random combinations of migration and spawning dates were used to simulate fish travel times and determine the energetic consequences of different thermal experiences during migration. An energy threshold criterion (4 kJ/g) was also imposed on survival and spawning success, which was used to determine how prevailing temperatures might select against certain migration dates and thermal experiences, and in turn, explain the selection for the current spawning phenology of the population. Scenarios of tributary use for thermal refugia under increasing water temperatures (1, 2, and 3°C) were also run to determine which combinations of migration dates, travel rates, and resulting thermal experiences might be most affected by energy exhaustion. As expected, when compared to observations, the model under existing conditions and energy use could explain the onset, but not the end of the observed spawning migration. Simulations of early migrants had greater energy loss than late migrants regardless of the river temperature scenario, but higher temperatures disproportionately selected against a larger fraction of early‐migrating fish, although using cold‐water tributaries during migration provided a buffer against higher energy use at higher temperatures. The fraction of simulated fish that exceeded the threshold for migration success increased from 58% to 72% as average seasonal river temperatures over baseline temperatures increased. The model supports the conclusion that increases in average seasonal river temperatures as little as 1°C could impose greater thermal constraints on the fish, select against early migrants, and in turn, truncate the onset of the current spawning migration.

## 1 INTRODUCTION

Energy use under prevailing temperatures is thought to shape the spawning migrations of Pacific salmon (*Oncorhynchus* spp.) because migration tactics and behaviors that result in excessive energy expenditure will be disadvantageous to survival (Crossin et al., 2004; Quinn, Peterson, Gallucci, Hershberger, & Brannon, 2002; Rand & Hinch, 1998). Water temperature controls the biological rates of salmon (Brett, 1995; Stewart & Ibarra, 1991), and higher water temperatures during upriver migration to spawn can elevate metabolic costs, delay spawning (Caudill et al., 2007; Keefer, Peery, Bjornn, Jepson, & Stuehrenberg, 2004), alter energy allocation to reproduction that can lead to prespawning mortality, or diminished reproductive success (Baigun, Sedell, & Reeves, 2000; Gilhousen, 1990; Major & Mighell, 1967; Schreck et al.,^1994^). Therefore, river temperatures that fish must endure during their upriver spawning migration may put bounds on upriver migration success, and in turn, help to shape the evolution of salmon spawning migrations.

Salmon enter freshwater with finite energy reserves that must be sufficient for the fish to migrate the given distance to spawn successfully under prevailing conditions. Because salmon are heterothermic and prefer cold to cool water (<16.5°C) and relatively high dissolved oxygen concentrations (>6 mg/L), prevailing seasonal temperature regimes can determine energy use by the fish and set temporal (and spatial) bounds on spawning migrations that often define salmon populations (Beauchamp, 2009; Bernatchez & Dodson, 1987; Quinn, Unwin, & Kinnison, 2000; Quinn et al., 2002). The effects of temperature on energy use and migration success may be particularly evident in salmon populations that undergo long and difficult migrations, especially when temperatures are beyond physiological optima (Bernatchez & Dodson, 1987; Crossin et al., 2004). Optimal physiological temperatures for adult Chinook salmon are ~16.5°C, and the (parabolic) physiological effects of temperature on either side of this optimal temperature are incorporated into the Wisconsin bioenergetics model for the species (Brett, 1995; Hanson, Johnson, Schindler, & Kitchell, 1997). Snake River fall‐run Chinook salmon (*Oncorhynchus tshawytscha*) migrate exceptionally long‐distances (>500 km) and initiate migration when summer temperatures are highest (Goniea et al., 2006; Keefer et al., 2004), indicating susceptibility to higher temperatures and metabolic constraints due to energy depletion during migration. Likewise, the fish may represent an ideal population to examine the extent to which temperature and metabolic demands may constrain migration success, and in turn, help shape the spawning phenology within the species.

Temperature and metabolic constraints can shape the onset of the spawning migration of salmon because a protracted migration may exhaust energy reserves causing early‐migrating fish to die before spawning (Rand et al., 2006). Quinn et al. (2002) demonstrated a shift in the spawning migration timing of Chinook salmon as result of the inadvertent selection for earlier‐migrating fish by hatcheries. Therefore, spawning migration timing by salmon appears to be (a) under genetic control, (b) can be influenced by external selection pressures that could (c) result in contemporary evolution of a population (within about three generations; Quinn et al., 2002; Stockwell, Hendry, & Kinnison, 2003). Given the species' demonstrated plasticity in phenology when transplanted to novel environments (Quinn & Unwin, 1993; Quinn et al., 1996, 2000) and the general importance of temperature to energy use by the species (Stewart & Ibarra, 1991), changes in the thermal regimes of rivers could alter energy use and spawning phenology.

This study examines how metabolic constraints and energy use may help shape the spawning phenology of Snake River fall‐run Chinook salmon by modeling the effects of temperature on energy use, and in turn, the migration and spawning success as defined by sufficient energy reserves (>4 kJ/g). Studies on the energy density of dead salmon on spawning grounds suggest the fish typically die once energy density falls below 4 kJ/g (Table 1). Crossin et al. (2004) was perhaps the first to propose the energy threshold (4 kJ/g) below which salmon death becomes imminent, whereas Rand et al. (2006) used this threshold to model migration success of sockeye salmon (*O*. *nerka*) in the Fraser River, and Hasler et al. (2012) used it to examine energetic consequences for Chinook salmon in the Puntledge River, BC, Canada. A proximate analysis of Snake River spring Chinook salmon during their spawning migration by Bowerman, Pinson‐Dumm, Peery, and Caudill (2017) also concluded the fish die once energy density approaches about 4 kJ/g. Thus, a total energy density of about 4 kJ/g is thought to provide a reasonable estimate of the lower energetic bound for successful migration and spawning in salmon (Hasler et al., 2012; Quinn et al., 2002), and higher temperatures may hasten the rate of the fish to this physiological threshold.

**Table 1:** Literature summary of estimates and assumed values for adult salmon energy at the start and end of the spawning migration.literature

Source	Stock/Species	Energy at river entry (kJ/g)	Energy at death (kJ/g)
Gende, Quinn, Hilborn, Hendry, and Dickerson (2004)	*Oncorhynchus gorbuscha*	5.9	3
Gende, Quinn, Wilson, Heintz, and Scott (2004)	*O. gorbuscha*	5.2	3.36
Penney and Moffitt (2014)	*O. mykiss*	6.7–8.9	4–4.5 (Kelt)
Hendry and Berg (1999)	*O. nerka*	6.6	2.95
Crossin et al. (2004)	*O. nerka*	9.5	4.47
Rand et al. (2006)^a^	*O. nerka*	~9.8	4
Mesa and Magie (2006)	*O. tshawytscha*	11.1	3.1
Hasler et al. (2012)^a^	*O. tshawytscha*	8.4 ‐ 9.6	4
Bowerman et al. (2017)	*O. tshawytscha*	11.7–12.1	3.6–4.1
This study^a^	*O. tshawytscha*	11.76	4
Jonsson, Jonsson, and Hansen (1991)	*Salmo salar*	5.5	3.84

a Values were assumed and used in modeling studies.

A bioenergetics model was used to estimate fish energy use from simulated thermal experiences observed for this federally listed population (NMFS, 1992). I examine which combinations of migration dates, travel rates, and thermal experiences result in excessive energy use and imminent mortality. The goal was to assess the extent to which recent (average) temperatures and energy depletion, or lack thereof, may help explain the current spawning phenology of the population. Model output is qualitatively compared to the observed timing of adult Snake River fall Chinook salmon returning to Bonneville Dam, OR. Because adult fish during upriver migration are known to use cold‐water tributaries in the lower Columbia River, presumably to use behavioral thermoregulation to conserve energy during migration, adding tributary use by the fish in the simulations enable an assessment of behavioral thermoregulation and its effect on fish thermal experience and energy use. At last, to assess the effect of energy depletion on spawning phenology under higher river temperatures, fish energy use was modeled as if daily river temperatures were, on average, 1, 2, and 3°C higher than current prevailing seasonal river temperatures, providing a relatively wide range in thermal experiences to evaluate the energetic benefits of different migration dates under varying thermal conditions and potential links between temperature, energy use, and the spawning phenology of the population.

### 1.1 Study area

**Figure 1:**
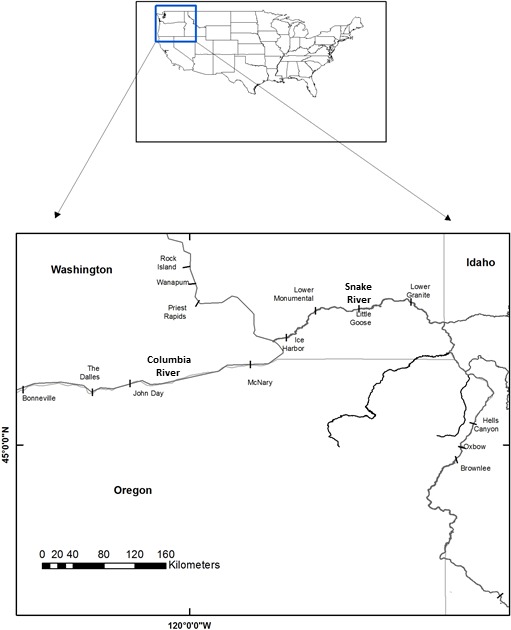
Map showing location of Columbia and Snake rivers in the Pacific northwest of the United States, and the dams and reservoirs (denoted by tick marks) experienced by Snake River fall Chinook salmon during their spawning migration from Bonneville Dam to Hells Canyon, Idaho

The study area encompassed 603 km of the Columbia and Snake rivers from Bonneville Dam (river kilometer 235 as measured from the mouth of the Columbia River) to the confluence of the Snake and Salmon rivers (rkm 838) that is centrally located in Hells Canyon (Figure 1). Migrating fish must pass eight large dams and reservoirs that range in length from about 36 km (The Dalles Reservoir) to 123 km (John Day Reservoir; Table 2) to reach Hells Canyon on the Snake River where about two‐thirds of the Snake River fall Chinook salmon population is thought to spawn (Groves & Chandler, 1999). Hells Canyon is a flowing stretch of river that extends from the top of Lower Granite Reservoir near Asotin, WA upstream to Hells Canyon Dam. This study considered the terminal location of the spawning grounds for Snake River fall Chinook salmon to be the central location at the confluence of the Snake and Salmon rivers.

**Table 2:** Summary statistics for ten‐year average daily water temperatures for the dams and reservoirs used in bioenergetics simulations of energy use by fall Chinook salmon during their typical migration period to the spawning grounds in Hells Canyon on the Snake River

Location (length; km)	Migration dates (8/1–10/10)		Spawning dates (10/10–12/31)	
	Range	Mean (°C, *SE*)	Range	Mean (°C, *SE*)
Bonneville (74.9)	16.7–21.4	19.8 (0.167)		
The Dalles (36.9)	17.6–22.4	20.5 (0.163)		
John Day (122.9)	17.8–22.4	20.5 (0.157)		
McNary (69.9)	17.2–21.3	19.8 (0.15)		
Ice Harbor (50.4)	16.7–21.2	19.7 (0.169)		
Lower Monumental (46.7)	16.8–21.4	19.5 (0.17)		
Little Goose (59.5)	17.1–20.9	19.1 (0.146)		
Lower Granite (63)	17.2–19.2	18.2 (0.066)		
Hells Canyon (78)	15.9**–**22.2	20.2 (0.221)	4.3–15.9	8.8 (0.384)

## 2 METHODS

### 2.1 Simulated migration and spawning dates

An important and intentional part of the design of this study was to include a wide range of possible migration dates and subsequent travel times and rates to the spawning grounds—because this may provide insight about observed (already preselected for) migration behaviors. Dates outside the typical range in passage (migration) dates at Bonneville Dam (1 June to 9 October; DART) and spawning dates in Hells Canyon (10 October to 1 April; Groves & Chandler, 1999) were used to simulate a wide range in migration dates and times (i.e., migration behaviors) for the simulated fish. Two constraints were imposed on the spawning dates of the simulated fish. First, the shortest time to travel to the spawning grounds was 7 days or a travel rate of about 85 km/day, which matched the maximum travel rates for this population reported by Keefer et al. (2004). Second, Chinook salmon are known to forego spawning until water temperatures fall below 16°C, which coincides with the temperature where egg survival increases rapidly (Boles, 1998; Jager, 2011). Migration and spawning dates were randomly drawn 100,000 times using a uniform distribution, and travel times were calculated as the difference in time between the randomly chosen migration and spawning dates, thus, travel rates (km/day) of the fish during simulations were assumed constant and were calculated by dividing the total distance to the spawning grounds (603 km; Table 2) by each simulated fish's travel time. Travel rates and dates of migration from Bonneville Dam are presented rather than travel time because travel rates can be more readily compared to other studies (e.g., Keefer et al., 2004).

### 2.2 Temperature exposure and scenarios

Water temperatures experienced by fish during migration are integral to the estimation of their energy expenditure. Data on historical water temperatures were obtained for each of the eight dams (DART) and used to represent the temperatures in each corresponding upriver reservoir. Average daily temperatures were calculated over a 10‐year period (2002–2012) as a measure of the current and prevailing temperature regime of the river. Average daily temperatures over the most recent 10 years for Hells Canyon were obtained from thermographs stationed throughout Hells Canyon (Idaho Power Company, unpublished) from 2002 to 2012. Correspondingly, water temperatures were a function of the simulated fish's travel rate and dates within each reservoir—although for a given date temperatures among the reservoirs did not markedly differ (Table 2).

Fall Chinook salmon migrating through the reservoirs above Bonneville and The Dalles dams can occupy cold‐water tributaries when main‐stem river temperatures become sufficiently high. Goniea et al. (2006) quantified the percent of fall Chinook salmon using cold‐water tributaries during upstream migration as well as the mean temperature differences from the main‐stem Columbia River temperatures for six tributaries. To account for whether or not a simulated fish used a cold‐water tributary and to evaluate the effect on different thermal experiences on the bioenergetics of the fish, the equation provided by Goniea et al. (2006) was used to calculate the probability of using a cold‐water tributary in the following manner:

(1) $$p\left(\text{tributary}\right)=\frac{6.558\cdot 10^{-7}\cdot e^{0.802\cdot \mathrm{Temperature}}}{100}$$

Each time a simulated fish passed one of the six tributaries (i.e., Wind River, Little White Salmon River, White Salmon River, Hood River, Klickitat River, and the Deschutes River) while migrating through the Bonneville and The Dalles reservoirs, the simulated fish had the opportunity to reside in a tributary from 1 to the total number of days of its predetermined travel time although the reservoir, assuming that it took a minimum of 1 day to travel the Bonneville or The Dalles reservoirs. The amount of time a fish could spend in a tributary was constrained by its predetermined total time spent in a reservoir and the number of cold‐water tributaries that were available in a reservoir. This was performed by randomly choosing a tributary from a multinomial distribution with the sample space *n *= 1 day less than the total days the fish spent in the reservoir, and the number of multinomial cell probabilities was equal to the number of available tributaries. If a simulated fish used a tributary, the temperature of the tributary was calculated by subtracting the average difference in temperature for each tributary (ranged from 1.5 to 6°C; Goniea et al., 2006) from the main‐stem Columbia River temperature used in the simulation. The functional form of the Goniea et al. (2006) equation is not logistic or constrained to result in values between 0 and 1, and when mean daily temperatures were >23°C yielded probabilities >1, in such cases, it is assumed that the probability of occupying a tributary in a simulation was equal to 1.

In addition to whether simulated fish used tributaries or not, three simple temperature scenarios were also evaluated by adding 1, 2, or 3°C to the daily river temperatures in the simulations to assess how changes in fish energy demands may change under warmer seasonal temperatures given current variability in daily temperatures. Such temperature scenarios also enabled an assessment of how the occupation of cold‐water tributaries may affect fish energy use under higher average river temperatures.

### 2.3 Bioenergetics simulation

This study uses bioenergetics modeling and simulation of 100,000 adult Chinook salmon (Stewart et al., 1983; Hanson et al., 1997; Stewart & Ibarra, 1991) to estimate energy use of fish based on thermal experiences while migrating from Bonneville Dam to Hells Canyon to spawn. The bioenergetics model consists of a series of log‐linear equations that use inputs on the fish's mass (*W*) and temperature at time *t* to predict the fish's mass (and energy density) at time *t *+* *1. The model reconciles the metabolic cost‐benefits associated with temperature‐ and allometric‐dependent physiological processes such as respiration (*R*), consumption (*C*), specific dynamic action (SDA), egestion (*F*), and excretion (*U*) using a mass‐balance framework (Stewart & Ibarra, 1991):

(2) $$W_{t+1}=W_{t}+C_{t}-\left({\text{SDA}}_{t}+R_{t}+U_{t}+F_{t}\right).$$

Inputs to the bioenergetics model included (a) the average daily temperatures for each date and reservoir over the simulated fish's intervening travel time to the spawning grounds, (b) food consumption, which was set to *C *=* *0, and (c) a starting mass. For all intents and purposes salmon returning from the ocean have no food available to them in freshwater rivers, and setting *C *=* *0 renders the values for SDA, *U*, and *F* equal to 0 and so these parameters did not contribute to energy use during simulations. I used a starting mass that approximated the mid‐point mass for Chinook salmon at Bonneville Dam of 7.9 kg (both sexes) for all simulations (Bowerman et al., 2017). To estimate energy density of the fish, *E*_D,*t*_, the “broken‐stick” regression equations for the energy density of Chinook salmon that is provided as part of the Wisconsin Bioenergetics model were used (Stewart & Ibarra, 1991):

(3) $$E_{D,t}=5,763+0.986\cdot W_{t}\;\text{when}\;W_{t}<4,000\;g$$

(4) $$E_{D,t}=7,598+0.527\cdot W_{t}\;\text{when}\;W_{t}>4,000\;\text{g}$$

The total energy content (*E*_C,*t*_; J) of a simulated fish at time *t* was determined by the product of the fish mass (*W*_*t*_) and energy density (*E*_D,*t*_; J/g). For example, a simulated fish with *W*_0_ = 7,900 g yields a total *E*_D,0_ = 11,761 J/g (or 11.76 kJ/g) and *E*_C,0_ = 92,914,270 J at *t *=* *0.

For a fish to spawn successfully a sufficient amount of the fish's initial energy reserves must be allocated to gonad formation, and thus, is not available to sustain metabolism for successful spawning. Research by Bowerman et al. (2017) indicated that male spring Chinook salmon allocated 2% and females 14% of their initial energy reserves to the formation of gonads. Based on sex ratios of adult Snake River fall Chinook salmon returning to Lower Granite Dam from 2004 to 2014, 60% of the adult returns were male (T. Cooney, NOAA fisheries, unpublished data). Thus, the weighted average energy allocation to gonad development (sexes combined) was about 6.8% of *E*_C,0_, yielding *E*_gonad_ = 7,433,142 J for a 7.9 kg fish. I assumed this energy allocation to gonadal development in all simulations, and subtracted *E*_gonad_ from the total energy content on date *t* to ensure energy allocation to the gonads for spawning.

The Wisconsin bioenergetics model uses fish weight to estimate fish energy density over time, and fish mass in the bioenergetics model declines over time when *C *=* *0. However, the bioenergetics model does not account for the physiological phenomenon of the fish replacing mass for water during migration when calculating the energy density of the fish. Therefore, when evaluated relative to the threshold, energy density of the fish was calculated using the fish's initial weight of 7.9 kg (*W*_0_ = 7,900 g; sexes combined), which assumes all mass lost during migration (as determined by the bioenergetics model) was replaced with water when assessing the energy density of the wet fish relative to the energy density threshold reported in the literature. Given energy allocation to the gonads and the replacement of mass for water, a simulated fish's available somatic energy density in time *t*,* E*_available,*t*_ was calculated as:

(5) $$E_{\mathrm{available},t}=\frac{E_{C,t}-E_{\mathrm{gonad}}}{W_{0}}$$

Evaluating *E*_available,*t*_ relative to the threshold energy density allows for the possibility of prespawning mortality with gonads and sufficient energy remaining to spawn as observed by Bowerman et al. (2017).

This study aims to assess the hypothesis that imposing a threshold of 4 kJ/g will help determine the temporal distribution and phenology of migration (passage) dates at Bonneville Dam. The available somatic energy density at time *t* was measured against the energy density threshold of 4 kJ/g below which the fish was assumed to die (Table 1). If energy loss and the threshold value of 4 kJ/g is a physiological constraint that determines fish survival and reproductive success, then certain migration dates (behaviors) that lead to excessive energy expenditure should be disadvantageous and therefore, selected against and not often observed in nature. Estimates of “survival” for the fish having sufficient energy to spawn is not intended to represent actual estimates of survival for Snake River fall Chinook Salmon. Rather the results of this study are best interpreted in a relative sense and within an evolutionary context, whereby certain migration dates and travel rates (or tributary uses) among those possible are selected against as determined by the resulting energetic consequences and the prevailing conditions. This study calculates the fraction of simulated fish having sufficient energy reserves (i.e., survivors) at spawning conditional on their date of migration from Bonneville Dam. These estimates are then compared qualitatively to the observed run‐timing of PIT‐tagged adult Chinook salmon that were tagged as juveniles in the Snake River. Congruence or lack thereof, between these observations should provide an assessment of the hypothesis that thermal—physiological constraints help to shape the spawning migration of the fish.

The distribution of observed migration dates of Snake River fall Chinook salmon at Bonneville Dam are qualitatively compared to the fractions of simulated fish having sufficient energy reserves at spawning. Observed migration dates at Bonneville Dam were based on PIT ‐tag detections of returning adults over a 10‐year period (2002–2012; obtained from PTAGIS website, http://www.ptagis.org) for natural‐ and hatchery‐produced fish that were tagged as juveniles and released upriver of Lower Granite Dam (Figure 1). The daily fractions of simulated fish that had sufficient energy reserves above the assumed energy threshold were calculated conditional on the date the fish migrated from Bonneville Dam. This provided a daily measure of the probability of having sufficient energy reserves to survive and spawn given the date the fish migrated from Bonneville Dam, *p*(spawn). Because simulations could be divided into fish that used tributaries and those that did not, *p*(spawn) was calculated for these groups of simulated fish, providing a relative measure of the energetic benefits of using cold‐water tributaries in the Lower Columbia River during upriver migration.

The model was run under scenarios of 1, 2, and 3°C increases in average seasonal temperatures, and *p*(spawn) was also calculated and graphically assessed for each temperature scenario for simulated fish that used and did not use tributaries during migration. The percent change in energy loss was calculated for each scenario, providing a relative comparison of the potential energetic consequences of migrating under higher average seasonal water temperatures. Perhaps most importantly, because this study used the literature‐derived threshold value of 4 kJ/g and a single benchmark average body weight of 7.9 kg for adult salmon, this study only provides an average estimate of the relative effects of temperature on energy density and selection pressures that may be imposed on the spawning migration of the fish. Although beyond the scope of this study, the model has the capacity to be adapted to evaluate differences in energy use among sexes, fish sizes, tributary use assumptions, and fish exposure to more sophisticated future climate change scenarios.

## 3 RESULTS

### 3.1 River temperatures and tributary use

The 10‐year average daily water temperatures were relatively similar among the study reservoirs and reach, yet these prevailing daily temperatures could exceed 21°C, a critical temperature threshold in salmon physiology, metabolism, and tributary use (Goniea et al., 2006; Stewart & Ibarra, 1991; Table 2). Temperatures for simulated migration dates ranged from 15.9 to 22.4°C and temperatures for spawning dates ranged from 3.9 to 18.1°C. Mean daily mid‐summer temperatures measured for Lower Granite Dam and Reservoir were slightly cooler than at other locations due to the release of cool water from an upstream storage reservoir on the Clearwater River for summer flow augmentation (Table 2, Figure 1; Cook et al., 2006).

**Figure 2:**
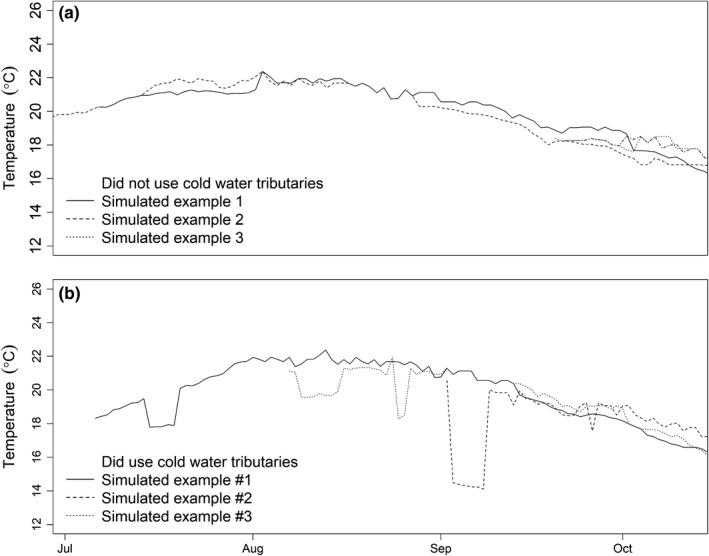
Examples of thermal experiences for simulated Snake River fall Chinook salmon based on 10‐year average daily temperatures that (a) did not and (b) did use one or more tributaries during upriver migration to spawn

**Figure 3:**
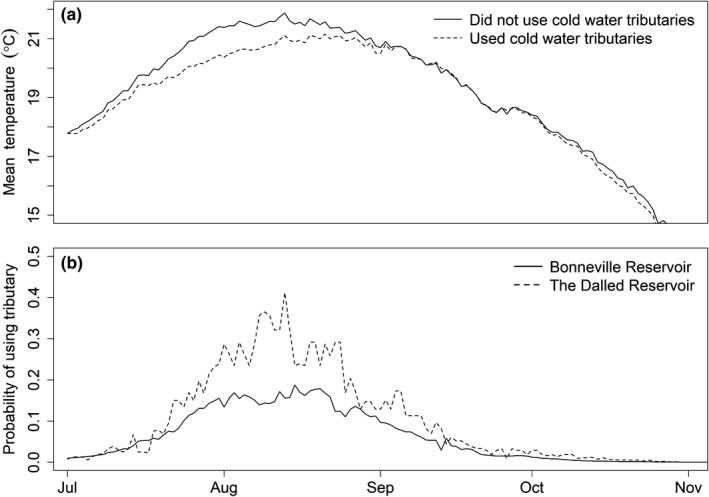
Time series of (a) the mean thermal experiences for simulated fish that used and did not use tributaries during their spawning migration, and (b) the probabilities of using a tributary (under the baseline +0°C scenario) based on Equation (1) by Goniea et al. (2006) and the 10‐year average daily temperatures for the Bonneville and The Dalles reservoirs on the main‐stem Columbia River

The thermal experiences of simulated fish were the result of the main‐stem river temperature, the temperature difference from main‐stem river temperatures for a tributary, and the probability of occupying a tributary as measured by Goniea et al. (2006). Time series of daily temperatures for the simulated fish demonstrated the effect of using tributaries on fish thermal experiences (Figure 2). Qualitatively, simulated thermal experiences appeared to be consistent with thermal experiences reported in the literature for upriver migrating salmon in the Columbia River (Keefer & Caudill, 2016). Based on the mean daily temperatures experienced by the simulated fish, using colder‐water tributaries reduced the mean temperature by about 0.5–1.1°C during peak summer temperatures. The effect of tributary use on a simulated fish's thermal experience depended on the time of year, the Columbia River temperature, and the Goniea et al. (2006) equation (Figure 3). When the Goniea et al. (2006) equation was applied to the daily temperatures in The Dalles Reservoir, there were higher daily probabilities of occupying the Deschutes River while in The Dalles Reservoir than occupying a tributary within the reservoir behind Bonneville Dam. This was the result of the nonlinear form of the Goniea et al. (2006) equation and the slightly warmer temperature in The Dalles Reservoir that were near the 21°C threshold in tributary use (Table 2; Goniea et al., 2006).

### 3.2 Migration dates, rates, and energy use

Given the range in conditions and dates considered across the simulations, there was also a relatively wide range in energy loss among the simulated fish. A 7.9‐kg fish at Bonneville Dam had an initial energy density of 11.76 kJ/g and a total energy content of 92.9 MJ, whereas depending on the migration and spawn date the energy density of simulated fish at spawning ranged from 0.38 to 10.23 kJ/g (median = 3.4 kJ/g). So the simulated fish lost as little as 13%, but as much as 97% of their initial energy reserves (median = 71%) during migration.

**Figure 4:**
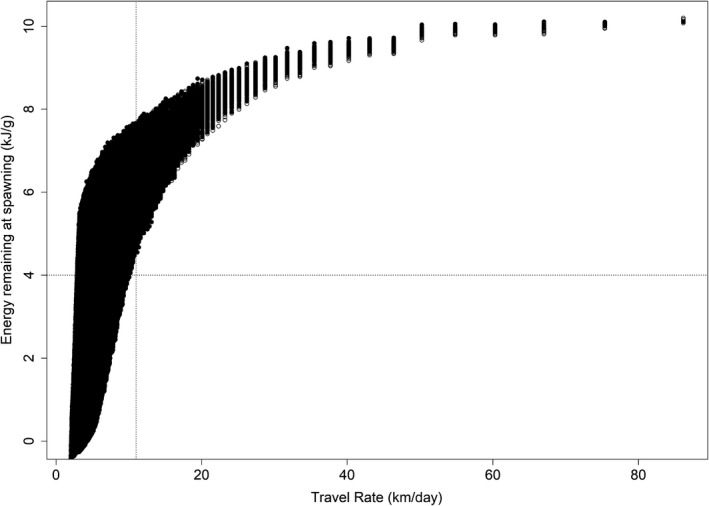
Bivariate relationship between Snake River fall Chinook salmon travel rates from Bonneville Dam to Hells Canyon, and their energy density at spawning during simulations of upriver migration

The randomly chosen migration and spawning dates over the given distance is what determined the simulated fish's travel rate from Bonneville Dam to Hells Canyon, and in part, its energy use during migration (Figure 4). The relation between energy use and travel rate was asymptotic, whereby the energy density of the simulated fish at spawning increased rapidly as the simulated fish traveled faster than >11 km/day. The relation between these variables reached an asymptote in travel rates at about 20 km/day, such that travel rates between 20 and 60 km/day were the slowest travel rates that provided the greater return on energy at spawning. These travel rates were also most similar to the interquartile range in observed travel rates reported for this population (Keefer et al., 2004).

**Figure 5:**
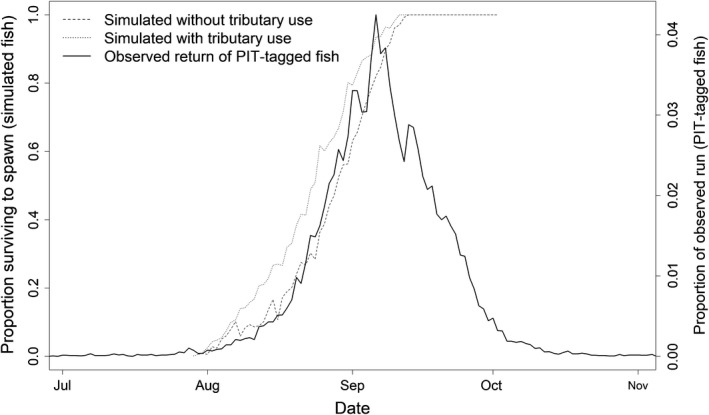
The fractions of simulated fish (given their day of migration from Bonneville Dam) that had energy densities greater than the theoretical 4 kJ/g threshold needed for sufficient energy to spawn. The fractions were estimated separately for simulated fish that used (dotted line) and did not use (dashed line) tributaries during their upriver migration. Also shown is the 10‐year average distribution of adult returns based on timing of detections at Bonneville Dam (thick solid line) for naturally‐produced Snake River fall Chinook salmon that were PIT‐tagged as juveniles

Early migration dates from Bonneville Dam or late spawning dates in Hells Canyon resulted in a relatively long travel time and slow travel rate to spawn. Of the fish that had travel rates <11 km/day, 67% had insufficient energy densities of <4 kJ/g to spawn. The use of cold‐water tributaries influenced the amount of available energy during migration and the fraction of fish having sufficient energy reserves to spawn. If a simulated fish used at least one cold‐water tributary during migration, then July 28th was the migration date from Bonneville Dam when at least one fish had sufficient energy reserves to spawn (i.e., >4 kJ/g). In contrast, if a simulated fish that did not use a tributary during upriver migration, then August 6^th^ was the first date of migration from Bonneville Dam when at least one fish had sufficient energy reserves to spawn (Figure 5), supporting the conclusion that using cold‐water tributaries as refugia during migration may provide early migrants with an energetic advantage over not using cold‐water tributaries.

### 3.3 Sufficient energy and observed adult returns

The fraction of simulated fish having sufficient energy reserves at spawning showed a strong concordance with the onset of the observed returns of adult fall Chinook salmon to the lower Snake River (Figure 5). The probability of having sufficient energy reserves primarily depended on: (a) the day of year the fish migrated from Bonneville Dam and (b) whether or not the fish used at least one tributary during its upriver migration. Likewise, the fraction of simulated fish having energy densities at spawning greater than the 4 kJ/g threshold value increased rapidly from late July to mid‐September, similarly, the fraction of the observed spawning population increased rapidly from late July to mid‐September. The dates that provided sufficient energy reserves at spawning for fish that used and did not use tributaries bracketed the observed dates of return for the PIT‐tagged Snake River fall Chinook salmon. The mode of the observed return of PIT‐tagged adult fish (10‐year average) was 6 September and the migration date at which 100% of the simulated fish had sufficient energy reserves to spawn was 13 September (whether fish used tributaries or not) Therefore, there was strong concordance between the observed onset of the spawning migration from Bonneville Dam and the fraction of simulated fish having sufficient energy reserves at spawning relative the theoretical threshold value of 4 kJ/g. This finding supports the hypothesis that a physiological energy floor in relation to thermal conditions may play an important role in shaping the spawning phenology of this fish population.

### 3.4 Increased temperature scenarios

**Figure 6:**
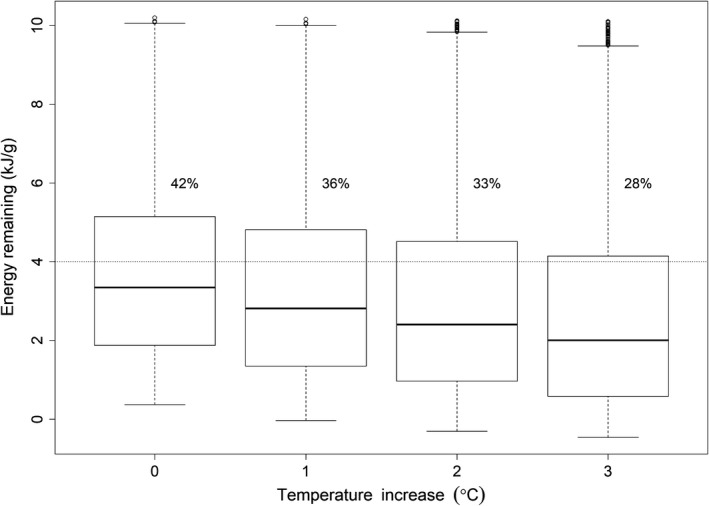
Box and whisker plots showing distribution of energy densities for simulated Snake River fall Chinook salmon during a migration under scenarios of higher seasonal river temperatures of +0, +1, +2, and +3°C. The plots show the medians (lines within the boxes), 25th and 75th percentiles (lower and upper boundaries of the boxes), and 5th and 95th percentiles (lower and upper ends of the whiskers) in the distributions of energy loss. Percentages above boxes represent the fraction of simulated fish that did not exceed the energy threshold of 4 kJ/g and presumably would not survive migration. The +0°C baseline scenario used the observed 10‐year average daily river temperatures (2002–2012) in each reservoir from Bonneville Dam to Hells Canyon, Idaho

**Figure 7:**
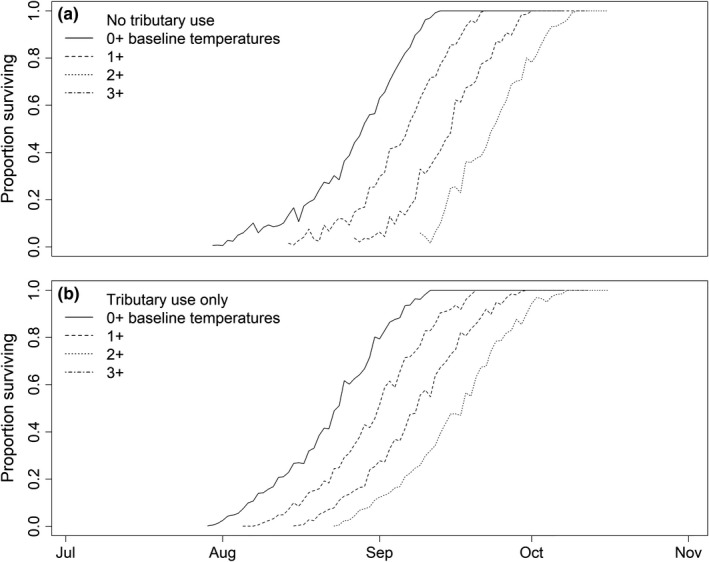
Fractions of simulated fish that had energy densities greater than the theoretical 4 kJ/g threshold needed for sufficient energy to spawn as function of the river temperature scenarios of +0, +1, +2, and +3°C for fish that (a) did not and (b) did use tributaries

Over the simulated range of dates (from June to dates <16°C), changing the 10‐year average daily river temperatures by 1, 2, or 3°C resulted in increased energy loss for the simulated fish during migration. There was a linear decline in the median energy remaining at spawning, and the fraction of simulated fish having sufficient energy reserves as average temperatures increased from base temperatures (Figure 6). For example, the median energy remaining at spawning declined by 0.43 kJ/g for every 1°C increase in the average seasonal river temperature over base temperatures. The consequence of higher energy use with increasing river temperature was a smaller fraction of fish having sufficient energy to spawn for a given day of year. For example, the passage dates at Bonneville Dam that provided a 50% chance of having sufficient energy at spawning for fish that did not use cold‐water tributaries was 15 August during the +0°C scenarios, 1 September during the +1°C scenario, 9 September during the +2°C scenario, and 20 September during the +3°C scenario. In contrast, the corresponding dates for fish that did use cold‐water tributaries were 28 July during the +0°C scenario, 5 August during the +1°C scenario, 15 August during the +2°C scenario, and 24 August during the +3°C scenario. Thus, as average seasonal temperatures increased the dates that could provide a similar chance of survival with sufficient energy reserves were delayed from 8 to 17 days later in the year (Figure 7). Also, as seasonal river temperatures increase, the dates from Bonneville Dam that afforded a >50% chance of having sufficient energy at spawning were 18–27 days earlier for the fish that did use tributaries.

## 4 DISCUSSION

Bioenergetics modeling of Snake River fall Chinook salmon during upriver migration provided a different, yet complementary approach to studies that have monitored the upriver migration of these fish (e.g., Caudill et al., 2007; Goniea et al., 2006; Keefer et al., 2004). Using simulation modeling and average prevailing water temperatures, I demonstrated how different migration dates, use of cold water refugia and higher prevailing temperatures might affect energy use and sufficient energy at spawning. As expected, energy use during migration was not a consideration for late spawning fish (i.e., beyond the mode of observed migration dates), but the fraction of early spawning fish with sufficient energy reserves at spawning in our simulations matched the onset of the observed migration of the PIT‐tagged fish returning to the Snake River, supporting the notion that temperature and thermal conditions during migration represents a significant factor that helps to shape this fish population's spawning phenology. Annual observations on thousands of radio‐tagged fall Chinook salmon in the Columbia and Snake rivers have demonstrated lower arrival probabilities to the spawning grounds with slower travel rates and greater exposure to higher water temperatures during migration (Caudill et al., 2007; Keefer et al., 2004). Although field studies on individually telemetered fish are powerful and informative, inferences are limited because maladaptive migration behaviors of unsuccessful migrants are inherently not observed (by virtue of natural selection) or excluded from analyses due to mortality (see Caudill et al., 2007). By contrast, computer simulation enabled the inclusion of disadvantageous migration behaviors (e.g., dates and tributary uses) to be assessed in relation to the observed phenology of the fish. Because this modeling study could quantify the fates of certain migration behaviors and tributary usages, the identification of different combinations of passage dates, migration rates, and thermal experiences that could define spawning success, or lack thereof, could be determined.

Perhaps this study's most remarkable finding was the strong concordance between the onset of the observed dates at which PIT‐tagged fish initiated migration and the fraction of simulated fish having sufficient energy at spawning. The similarity in timing and increase between observations and simulations is unlikely to be spurious, with the frequency of a successful behaviors (e.g., migration timing) giving rise to frequency of behaviors observed in nature. This finding is not wholly unexpected given that adaptation of phenology by salmon is demonstrated in nature by seasonally distinct groups of fish that spawn at different elevations in the same river (O'Malley et al., 2013), or artificially via inadvertent selection by hatcheries (Quinn et al., 2002) and introductions to new habitats (Quinn et al., 2000). Many selective factors may interact with thermal regimes that can alter survival and help to shape the spawning season of salmon (Quinn et al., 2002), but warmer temperatures alter metabolic efficiency and the rate of senescence, and so temperature can certainly set a lower bound on migration and reproductive success (Hasler et al., 2012; van den Berghe & Gross, 1989).

The relation between energy density at spawning and the travel rates of simulated fish is supported by empirical studies on Snake River fall Chinook salmon. Interquartile ranges in travel rates from about 20 to 60 km/day have been reported for fall Chinook salmon passing main‐stem dams on the Columbia and Snake rivers (Keefer et al., 2004). The inter quartile range in travel rates measured for this population (Keefer et al., 2004) were near the asymptote in simulations travel rates and energy at spawning, suggesting that the migration dates and travel rates observed in nature may be optimal for energy at spawning. Geist, Abernethy, Blanton, and Cullinan (2000) used electromyogram telemetry to develop relationships between swimming speed and energy use for fall Chinook salmon. To provide context for their results, they estimated energy use during migration to Hells Canyon and concluded that migrating fall Chinook salmon having travel rates <11 km/day (i.e., travel times > 55 days) would be unlikely to make it to the spawning grounds or spawn successfully. Fish having travel rates much slower than those reported by Keefer et al. (2004) and cautioned against by Geist et al. (2000) had energy losses less than the posited energy content threshold of 4 kJ/g (Crossin et al., 2004), supporting the notion that fish traveling much slower than 11 km/day or taking much longer than 55 days would be less likely to spawn successfully—or observed in nature.

The simulated fish that had the earliest migration date from Bonneville Dam always had relatively slow travel rates to spawn because the spawning date (10 October) was dictated by when river temperatures in Hells Canyon declined to <16°C—the temperature threshold for spawning and egg survival (Boles, 1998; Groves & Chandler, 1999; Jager, 2011). For these fish, their temperature exposure and energy loss were always relatively high, having 100% probability of falling below the energetic threshold of 4 kJ/g and dying before their spawning date. In contrast, simulated fish that migrated later in the year under cooler temperatures had generally faster travel rates and were exposed to lower average temperatures that reduced energy loss and increased the chances of survival to spawn. For fish arriving at Bonneville Dam at the onset of the migration, the use of cold‐water tributaries could be considered critical for offsetting the energetic demands of higher water temperatures, whereas for fish that migrate later, occupying colder‐water tributaries may provide little energetic benefit.

Simulated fish that migrated from Bonneville Dam sufficiently late in the season always had enough energy reserves to spawn, and 100% survived to spawn when migrating later than 13 September (Figure 5). Therefore, the biological limit of 4 kJ/g to sustain biological function under prevailing temperatures could not explain the end of the observed spawning migration. Other factors such as subsequent selection pressures and evolutionary feed‐backs resulting from the poor survival of eggs and fry as a consequence of late spawning are likely mechanisms that could explain the end of the spawning migration (Brannon, 1987; Manhard, Joyce, & Gharrett, 2017; Rand et al., 2006). The inability for energy use to explain the end of the observed spawning migration was not an unexpected result. This study is limited to the extreme case of Snake River fall Chinook salmon, and applying a similar approach to spring and summer runs of Chinook salmon may provide a fuller explanation of how temperature, migration dates, and energy use assist in the temporal and spatial diversity in the spawning phenology of Chinook salmon.

There are many caveats and assumptions to consider because this study used a relatively simple travel time model and a commonly used bioenergetics model to assess the effects of higher water temperatures on the “average” Chinook salmon migrating through a highly variable and geologically diverse environment. I used an assumed adjustment for the replacement of mass for water and average gonad energy allocation when applying the bioenergetics model to upriver migrating Chinook salmon. This was performed to account for the fact that mass‐water replacement is not considered by the bioenergetics model as well as to facilitate comparison to the returning PIT‐tagged fish with unknown sexes. Also, size‐ and temperature‐dependent activity coefficients and energy densities that are built into the Wisconsin Bioenergetics model are uncertain and could be improved (Ney, 1993). This study can make few statements about the conditions that fish truly experience when traveling through the Columbia and Snake rivers (and dams) to spawn. Nonetheless, by incorporating average temperature increases and probabilities of occupying cold‐water tributaries, this study considered a relatively wide range of thermal experiences and outcomes on the energy budget of the average Chinook salmon during migration to the Snake River. Fish may also experience temperature‐disease interactions (Ray, Perry, Som, & Bartholomew, 2014), predation by humans and pinnipeds (Z. Penney, Columbia River Intertribal Fish Commission, personal communication), experience the effects of flow and fallback after ascending dams that can vary due to dam operations (Caudill et al., 2007; Salinger & Anderson, 2006). These factors can affect fish survival and energetic status over time during upriver migration, and in turn, help shape the observed spawning phenology of the fish. Because temperature was the main driver of energy use in the model and model predictions did not markedly depart from observations at the onset of the migration dates from Bonneville Dam, the model estimates of energy use under base temperatures were likely sufficient to assess the general hypothesis that temperature‐dependent constraints on energy use helps to shape the spawning phenology of the fish.

Because water temperature determines the rate at which individual fish will reach the energetic floor (4 kJ/g), energetic demands required to undergo long‐distance migration (e.g., 500 km) reduces the energetic “margin” of long‐distance migrants compared to salmon that migrate much shorter distances (Rand et al., 2006). This would suggest that fish will have less energetic capacity in warmer water, and that selective pressures due to higher temperatures may differentially be imposed. It is notable that this study showed that temperature‐dependent selection against early migrants could occur over a relatively small increase in temperature (~1°C) over current prevailing river temperatures. Given that energy use was modeled for a long‐distance migrant (>500 km) at temperatures that were near or above the known physiological optima for the fish (Goniea et al., 2006; Hanson et al., 1997), energy use during the spawning migration should presumably play a very limited role in shaping the evolution of spawning migration timing for populations that travel shorter distances under cooler conditions—although more coastal populations may require lower lipid energy stores for migration (Table 1).

It is important to mention that this study considered simple temperature scenarios—not scenarios of river conditions predicted under future climate change. Fish migration was simulated using a set of very simple increases in temperature over and above prevailing seasonal temperatures, and likewise this study does not account for the myriad of potential changes in precipitation, timing of snow melt and subsequent changes in river flows, or oceanic effects on energetic status and maturation of returning adults that may occur prior to spawning under future climate change (Crozier & Hutchings, 2014; Urawa et al., 2016). Based on metabolic power (adenosine triphosphate re‐synthesis per unit of time) calculations with temperature, Martin, Nisbet, Pike, Michel, and Danner (2015) concluded that ignoring constraints on metabolic power may result in as much as a 60% underestimation of the effect of temperature on salmon migration costs. From this perspective, because the Wisconsin bioenergetics model does not explicitly account for metabolic power demand, but rather uses a mass‐ and temperature‐dependent coefficient for activity on fish respiration (Stewart & Ibarra, 1991), conclusions from this modeling study about the effects of temperature on salmon and energy use to spawn may be conservative.

## CONFLICT OF INTEREST

None declared.

## AUTHOR'S CONTRIBUTION

As the sole author of this study, I take full responsibility for its content; however, this study was certainly improved with the help and suggestions of others, please see the acknowledgments.
